# Supplementary material for: In Vitro Screening of Chicken-Derived Lactobacillus Strains that Effectively Inhibit Salmonella Colonization and Adhesion
Source: Foods. 2021 Mar 9;10(3):569. doi: 10.3390/foods10030569 (PMC7998290; doi:10.3390/foods10030569)
Supplement: Supplementary file 1 [file foods-10-00569-s001.pdf]

Supplementary- Biochemical identification of *Lactobacillus*

1 physiological and biochemical identification of lactic acid strains

1.1 Morphology of isolated strains

The morphological description of the selected *Lactobacillus* is shown in Table S1 of supplementary materials. It can be seen from Table S1 that the colony color is mainly gray white, milky white and surface white, the shape is mainly round, the surface is smooth and moist, the texture is uniform, easy to pick; Under the microscope, the individual shape of the plant was observed as long and thin rod, thick and short rod, ball and so on.

1.2 Gram staining results of isolated strains

120 samples of chicken intestines were collected from different chicken slaughterhouses. The samples were mixed, and 25g samples were added to 225ml normal saline. The samples were subjected to constant temperature oscillation at 37°C for 30min. The mixed samples were diluted into 10^{-3},10^{-4},10^{-5},10^{-6},10^{-7} in turn, and then coated on MRS solid medium. After coating, the samples were cultured in an inverted incubator at 37°C for 48h. After the culture, the typical *Lactobacillus* colony form and the suspected *Lactobacillus* with obvious sour taste were selected and transferred in MRS liquid medium. After transfer, the *Lactobacillus* was cultured in 37°C incubator for 48h. After the culture, again coated. Typical 107 strains of lactic acid bacteria were selected for Gram staining and timely preservation . After solid medium streaks were used to pick out suspected single colonies as in Figure S1, and then transferred to MRS liquid medium twice for culture, 98 strains of suspected lactic acid bacteria were screened out. After Gram staining, 89 strains turned purple after gram staining. After microscopic examination, it was found

that the strains turned purple after gram staining were spherical, short chain shape and rod shape. From this, 89 strains were identified as suspected lactic acid bacteria.

1.3 physiological and biochemical identification

Physiological and biochemical identification of *Lactobacillus*. According to the Identification Manual of *Lactobacillus* compiled by Ling Daiwen et al. (1999), nitrate reduction test, glucose gas production test, exercise test.

(a) Gelatin liquefaction test. One ring of suspected strains was inoculated in gelatin medium and cultured at 37 ℃ for 24 hours to observe the solidification of the medium.

（b）Indole test. One ring of suspected strains was inoculated into tryptophan medium, and cultured at 37 ℃ for 48h at constant temperature. Two drops of indole reagent were added to observe the color change of the upper solution of the medium.

(c) Exercise test. In MRS broth medium, 0.4% agar powder was added to prepare semi-solid medium, in which one ring of suspected strains was inoculated, and cultured in biochemical incubator at 37 ℃ for 48h to observe the growth of bacteria in semi-solid medium.

1.4 Results of physiological and biochemical experiments of lactic acid bacteria

A total of 89 strains of *Lactobacillus* were screened out, and the biochemical test results of 36 strains were listed in the Table S2 of supplementary materials. The 36 strains of *Lactobacillus* with positive gram staining, negative catalase test and no spores were able to form calcium-soluble circles around the colony of MRS solid medium containing $CaCO_3$. The colonies were round in shape, with neat edges, slightly

convex or flat in the center, white, milky yellow or nearly transparent in color, smooth and shiny on the surface, and 0.5-2mm in size.The 39 strains of *Lactobacillus* isolated from the samples with positive gram staining, negative catalase test and no spores were able to form calcium-soluble circles around the colony of MRS solid medium containing $CaCO_3$. The colonies were round in shape, with neat edges, slightly convex or flat in the center, white, milky yellow or nearly transparent in color, smooth and shiny on the surface, and 0.5-2mm in size. The results showed that each strain could produce acid from glucose without motility, without producing hydrogen sulfide or indoles, without liquefying gelatin or reducing nitrate, which was consistent with the basic characteristics of lactic acid bacteria. Other physiological and biochemical tests, such as glucose gas production test, acid and salt tolerance test, development temperature test and arginine hydrolysis test, have different results.

2 Growth characteristics of Lactobacillus

2.1 Growth curve of Lactobacillus

The suspected strains were inoculated into MRS broth medium, cultured at 37 ℃, and sampled every 2 h. Their OD values were measured at 600 nm wavelength, and the relation between culture time and strain growth was observed to draw a growth curve.

Figure S2 shows the growth of 24 LAB isolates within 48 hours. In **Figure S2, a, b** and **c** show the growth results of 8 strains of LAB,

respectively. **a**-The growth curve of strains LAB1, LAB4, LAB7, LAB12, LAB14, LAB16, LAB22, LAB24; **b**-The growth curve of strains LAB26, LAB31, LAB34, LAB35, LAB44, LAB53, LAB54, LAB56, LAB60; **c**- The growth curve of strains LAB64, LAB65, LAB69, LAB70, LAB72, LAB73, LAB76, LG. In general, the growth of 24 strains of LAB showed S-shaped growth characteristics, 0–2 h was the delay period, 3–18 h was the logarithmic growth period, and the growth reached the stable stage after 18 h. LAB16, LAB24, LAB34, LAB69, LAB76, and LG grew rapidly.

2.2 Acid production capacity test of Lactobacillus

The suspected strains were inoculated into the MRS broth culture medium, the pH of which was measured every 2 h at 37 °C, to determine the acid production ability of the suspected strains.

Figure S3 shows the change in acid production capacity of 24 LAB strains within 48 hours. a- The growth curve of strains LAB1, LAB4, LAB7, LAB12, LAB14, LAB16, LAB22, LAB24; b-The growth curve of strains LAB26, LAB31, LAB34, LAB35, LAB44, LAB53, LAB54, LAB56,; c- The growth curve of strains LAB60, LAB64, LAB65, LAB69, LAB70, LAB72, LAB73, LAB76, LG. In general, the 24 strains had strong acid production capacity; however, there were some differences. The pH value of LAB44 and LAB54 decreased from 6.08 to 4.19 after 8 hours of growth, which were the two fastest acid-producing strains; and the pH value of LAB44 and LAB54 decreased from 6.08 to 4.19 after 8

h of growth, and the pH of LAB69 and LAB70 became 3.45 after 48 h. The pH value of LAB69 and LAB70 changed to 3.45 after 48 h, which were the two strains with the strongest acid-producing ability.

2.3 Acid and alkali resistance test results of Lactobacillus strains

The acidity of the MRS broth medium was adjusted by using HCl solution at a pH of 2.0, 3.0, 6.0, 7.0, and 8.5. Each strain was inoculated into the MRS broth medium with different acidity gradients (thrice for each gradient). A control (pH 7.2) was cultured at 37 °C for 48 h to observe the strain growth.

Figure S4 shows the growth of 24 strains of LAB under 5 different pH conditions for 48 h. a- The growth curve of strains LAB1, LAB4, LAB7, LAB12, LAB14, LAB16, LAB22, LAB24; b-The growth curve of strains LAB26, LAB31, LAB34, LAB35, LAB44, LAB53, LAB54, LAB56; c- The growth curve of strains LAB60, LAB64, LAB65, LAB69, LAB70, LAB72, LAB73, LAB76, LG. Under the conditions of 0 h and pH 2.0, the growth of 24 strains of LAB was determined as a blank control group. Compared with the control group, the growth of 24 strains showed an upward trend. The results showed that 24 strains of LAB grew slowly under the conditions of pH 2.0 and pH 3.0, and grew better in neutral and alkaline conditions than in acidic environment, and the best growth was at pH 7.2.

2.4 Lactobacillus surface characteristics

2.4.1 Determination of self-polymerization ability of Lactobacillus strains

Lactobacilli were cultured in MRS medium for 24 h, collected by centrifugation (4500 ×*g*; 15 min), washed twice with PBS (pH 7.2), and re-suspended in PBS. The number of bacteria was adjusted to 10^8 CFU/mL, and 4 mL of the same cell suspension was placed into a 5 mL centrifuge tube at room temperature. Auto-polymerization was determined at 0, 2, 4, 6, and 8 h. At each time point, we collected 0.5 mL supernatant, added it to 1.5 mL PBS, mixed well, and measured the absorbance at 600 nm. PBS was considered the blank control. The agglutination rate was calculated as:

$$A\,(\%) = \frac{A_0 - A_t}{A_0} \times 100 \quad (1)$$

(1): A_0: absorbance at 600 nm at the initial time point; A_t: absorbance at different time points.

2.4.2 Determination of surface hydrophobicity of Lactobacillus strains

The surface hydrophobicity of strains was determined by the microbial adhesion to hydrocarbons method. The bacterial culture was centrifuged at 4500 ×*g* for 15 min, eluted twice with PBS (pH 7.2), and re-suspended in PBS. The number of bacteria was adjusted to 10^8

CFU/mL, and 3 mL bacterial suspension and 0.5 mL xylene were mixed for 60 s. Then, after 10 s of pause, the mixture oscillated for 60 s, and placed at room temperature for 60 min for stratification. The lower aqueous phase was collected, and the buffer was used as the blank control, to measure and record absorbance at a 600 nm. Surface hydrophobicity was calculated as:

$$H (\%) = \frac{A0 - A}{A0} \times 100 \quad (2)$$

(2): A_0: absorbance at 600 nm before mixing with xylene; A: absorbance at 600 nm after mixing with xylene.

2.4.3 Determination of the copolymerization ability of Lactobacillus strains and pathogenic bacteria

Lactobacillus strains were cultured in MRS medium for 24 h, separated by centrifugation (4500 ×*g*, 15 min), and washed twice with PBS (pH 7.2). Both th*e* LAB *a*nd the SAL were re-suspended in PBS, and their number was adjusted to 10^8 CFU/mL. We collected 2 mL suspensions of the LAB and SAL (LAB+SC85, LAB+SE05) into test tubes, shook them with a vortex mixer for 10 s, and measured their light absorption at 600 nm after 0, 2, 4, 6, and 8 h. The agglutination rate of the pathogenic bacteria was calculated based on the following formula:

$$A (\%) = \frac{A_0 - A_{\mathrm{mix}}}{A_0} \times 100 \quad (3)$$

(3) : A_{mix}: absorbance value of the pathogenic bacteria and bacteria mixture (LAB+SC85, LAB+SE05) tested at each time point; A_0:

absorbance value of the mixture at the initial time point.

2.4.4 Surface active molecule analysis of Lactobacillus strains

The *Lactobacilli* were activated for three consecutive generations using 5 mol/L of LiCl. Following this, they were placed into 10 mL MRS medium, cultured for 18 h, and centrifuged (4500 ×*g*, 10 min, 4 °C). The precipitates were washed twice with sterilized PBS (4 °C, pH 7.2), suspended in 2 mL of 5 mol/L LiCl, and cultured for 60 min in an incubator shaker (200 r/min) at 37 °C. Following this, the bacteria were collected by centrifugation (4500 ×*g*, 15 min) and resuspended in PBS, and their number was adjusted to 10^8 CFU/ml. We collected 2 mL suspension of the LAB and SAL (LAB+SC85, LAB+SE05) into test tubes, shook them with a vortex mixer for 10 s, and measured their light absorption at 600 nm after 0, 2, 4, 6, and 8 h. The agglutination rate of the pathogenic bacteria was calculated using the following formula:

$$A\ (\%) = \frac{A_0 - A_{\mathrm{mix}}}{A_0} \times 100 \quad (4)$$

(4) : A_{mix}: absorbance value of the pathogenic bacteria and bacteria mixture that was tested at each time point; A_0: absorbance of the mixture at the initial time point. The specific operation of the NaI method was the same as the LiCl method.

Figure S5 shows the test results of the self-aggregation ability of 24 LAB strains within 8 hours. a- The growth curve of strains LAB1,

LAB4, LAB7, LAB12, LAB14, LAB16, LAB22, LAB24; b-The growth curve of strains LAB26, LAB31, LAB34, LAB35, LAB44, LAB53, LAB54, LAB56, LAB60; c- The growth curve of strains LAB64, LAB65, LAB69, LAB70, LAB72, LAB73, LAB76, LG. The self-aggregation ability of 24 LAB increased within 8 hours, but there were some differences. LAB14, LAB31, LAB35, LAB70, and LAB73 had the strongest self-polymerization ability. As shown in Figure S6, the agglutination of *Lactobacillus* to two *Salmonella* strains after the LiCl treatment was not consistent. Lithium chloride can destroy S-layer protein that destroys the surface of *Lactobacillus*; however, the copolymerization ability of some *Lactobacillus* strains treated with LiCl was higher than that of the untreated strain. The S-layer protein may have a masking effect on the polysaccharide adhesion site. As shown in Figure S7, the agglutination effect of *Lactobacillus* treated with $NaIO_4$ on two *Salmonella* strains was not consistent. The purpose of treating *Lactobacillus* with $NaIO_4$ was to destroy their surface polysaccharides. Increased *Lactobacillus* agglutination rates indicate that the possible adhesion site is the polysaccharide.

Figure S8 shows the results of the hydrophobicity of 24 LAB isolates within 24 hours. The hydrophobicity of 24 strains of LAB was different. LAB1, LAB12, LAB24, LAB26, LAB31, LAB69, LAB70, LAB72, LAB73, LAB76, LG, which had a hydrophobic ratio higher than 60%, showed strong hydrophobicity. The results showed that different LAB strains have different hydrophobicity. This is consistent with the results of Tuo et al. (2013).

2.5 Comparison of tolerance of LAB in artificial intestinal fluid

In this study, 0.3% (w/v) bovine bile salt and 1 g/L trypsin were used to simulate human intestinal fluid. A 100 μL bacterial suspension was prepared from 24 suspected *Lactobacillus* strains, cultured in MRS medium at 37 °C for 24 h, and activated for one generation. The activated strains were inoculated into 2% of the sterilized MRS medium for 24 h. The bacteria were centrifuged at 12,000 r/min for 2 min, collected, and resuspended in 2 mL sterile normal saline. We extracted 0.5 mL LAB suspension and mixed it with 4.5 mL simulate human intestinal fluid. The negative control was mixed with normal saline. After labeling, the culture was incubated in a shaker at 37 °C and 100 r/min for 4 h. Next, we inoculated 500 μL of the treatment solution above into 5 mL of the culture medium, incubated it in a shaker at 37 °C and 100 r/min for 8 h, measured the absorbance at 600 nm, and calculated the survival rate according to the following formula:

Survival rate (%) = $\frac{B}{A}$×100 (5)

(5) : *A*: absorbance of normal saline; *B*: absorbance of various treatment solutions.

Figure S10 shows the tolerance of 24 strains of LAB to different components in simulated artificial gastric juice. a-The tolerance of 24 strains of LAB to pepsin in artificial gastric juice; b is the tolerance of 24 strains of LAB to trypsin in artificial gastric juice. Eleven strains of LAB showed strong tolerance to pepsin, and 15 *Lactobacillus* strains were highly tolerant to trypsin, with survival rates higher than 50% after the treatment

(Figure 10). Overall, 6 lactic acid bacteria strains, namely LAB4, LAB12, LAB24, LAB69, LAB76, LG, were highly tolerant to both pepsin and trypsin, i.e., the artificial gastrointestinal environment.

2.6 *Electrophoresis experiment of Lactobacillus*

Based on the aforementioned experiments, we screened 16 *Lactobacillus* strains with typical physiological and biochemical characteristics, such as acid-base, drug, and bile salt resistance. 27F and 1492r primers and 16S high fidelity enzyme were used for PCR amplification and sequencing identification (Henan ShangYa Biotechnology Co., Ltd.), and water was used to replace *Lactobacillus* as a negative control. PCR products were analyzed by gel electrophoresis using 1% agarose (Lonza Rockland, Inc., Rockland, ME, USA) and a 2000 bp DNA ladder (Solarbio, Beijing, China) as a molecular weight marker.

After PCR amplification, the band fragment shown in the figure is 1500 bp, which should appear after the 16S amplification of lactic acid bacteria. The electrophoretic bands were clear and free of heterobands, indicating that the amplified PCR products could be sequenced (Figure 5-1 and 5-2).

Table S1. Colony characteristics and Bacterial forms of different strains.

Strains	Colony characteristics	Bacterial morphology
LAB 4	Small colony, raised, gray white, moist, neat edge, round, moist and smooth, easy to pick	Long G^{+} bacilli, in pairs or in chains+
LAB21	Small colony, slightly raised, milky white, neat edge, moist and smooth, flat and round, easy to pick	G^{+} slender bacilli, in pairs or in chains
LAB 35	The colony is small, slightly raised, milky white, the edge is neat, moist and smooth, flat and round, and the surface is rough	G^{+} club, single or in pairs
LAB44	Colony gray, translucent, smooth surface	G^{+} spherical, short chain arrangement
LAB53	Large colony, raised, white, neat edge, smooth and moist surface, round, easy to pick	G^{+} slender bacilli, in pairs or in chains
LAB 69	Small colony, slightly raised, gray white, neat edge, moist and smooth, flat and round, rough surface	G^{+} thick short rod, single or in pairs
LAB72	Colony is gray white, flat, translucent, round, with neat edge	Long G^{+} bacilli, in pairs or in chains
LAB76	Colony is gray white, flat, translucent, round, with neat edge	Long G^{+} bacilli, single or double chain

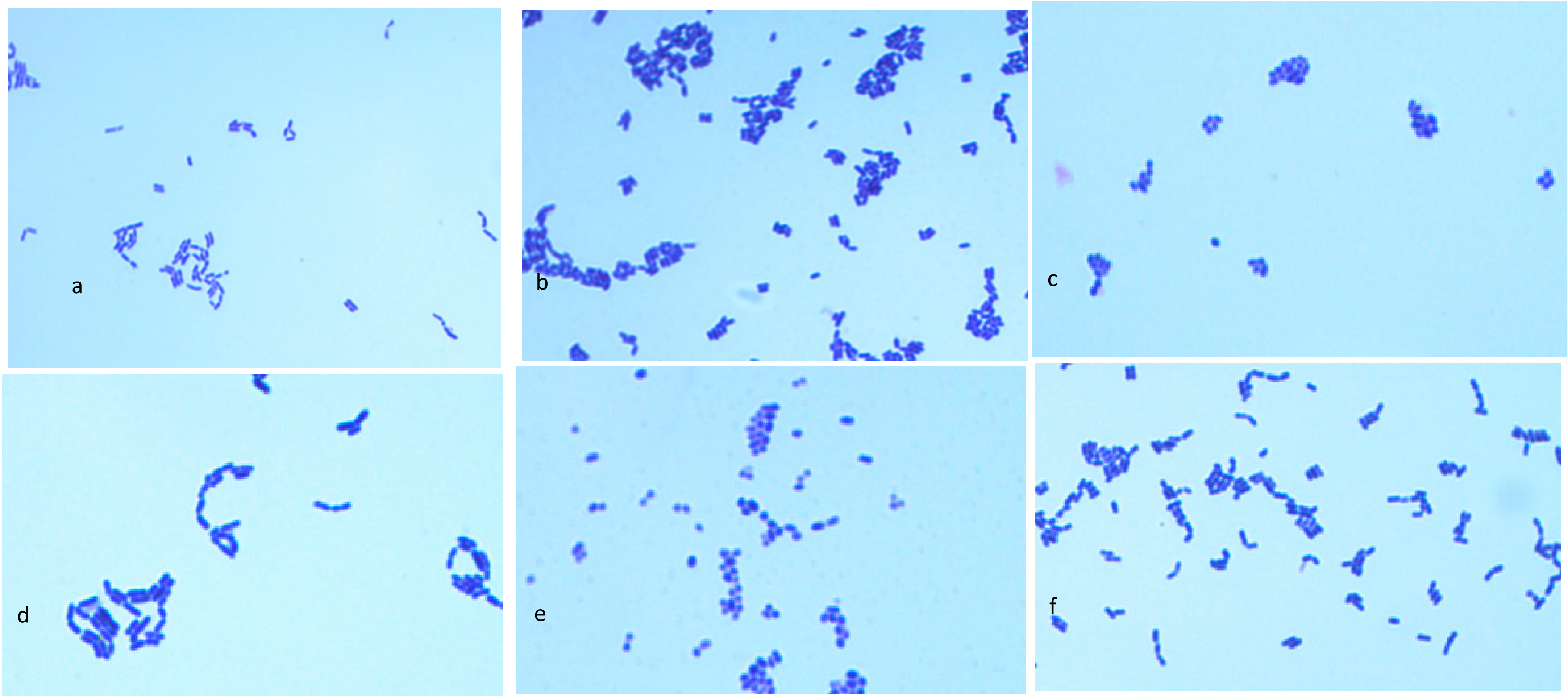

Figure S1. Gram staining results of strains isolated from *Lactobacillus*.

Note: After microscopic examination, it was found that the gram stain changed to purple, with spherical, short chain and rod-shaped morphology. In **Figure 1**, the pictures marked with **a, b** and **f** are short chain shaped, those marked with **c** and **e** are short chain shaped, and picture **d** is rod-shaped.

Table S2. test results of physiological and biochemical characteristics of *Lactobacillus*.

**Detection index** \ **Strains**	**1**	**4**	**7**	**12**	**14**	**16**	**20**	**21**	**22**	**23**	**24**	**26**	**30**	**31**	**32**	**34**	**35**	**36**
Gram staining	+	+	+	+	+	+	+	+	+	+	+	+	+	+	+	+	+	+
H_2O_2	—	—	—	—	—	—	—	—	—	—	—	—	—	—	—	—	—	—
Mobility	—	—	—	—	—	—	—	—	—	—	—	—	—	—	—	—	—	—
Hydrogen sulfide test	—	—	—	—	—	—	—	—	—	—	—	—	—	—	—	—	—	—
Indole test	—	—	—	—	—	—	—	—	—	—	—	—	—	—	—	—	—	—
Gelatin liquefaction test	—	—	—	—	—	—	—	—	—	—	—	—	—	—	—	—	—	—
Glucose acid production test	+	+	+	+	+	+	+	+	+	+	+	+	+	+	+	+	+	+
Glucose gas production test	—	—	—	—	—	—	—	—	—	—	—	—	—	—	—	—	—	—
Nitrate reduction	—	—	—	—	—	—	—	—	—	—	—	—	—	—	—	—	—	—

**Detection index** \ **Strains**	**44**	**50**	**53**	**54**	**56**	**58**	**60**	**61**	**63**	**64**	**65**	**67**	**69**	**70**	**71**	**72**	**73**	**76**
Gram staining	+	+	+	+	+	+	+	+	+	+	+	+	+	+	+	+	+	+
H_2O_2	—	—	—	—	—	—	—	—	—	—	—	—	—	—	—	—	—	—
Mobility	—	—	—	—	—	—	—	—	—	—	—	—	—	—	—	—	—	—
Hydrogen sulfide test	—	—	—	—	—	—	—	—	—	—	—	—	—	—	—	—	—	—
Indole test	—	—	—	—	—	—	—	—	—	—	—	—	—	—	—	—	—	—
Gelatin liquefaction test	—	—	—	—	—	—	—	—	—	—	—	—	—	—	—	—	—	—
Glucose acid production test	+	+	+	+	+	+	+	+	+	+	+	+	+	+	+	+	+	+
Glucose gas production test	—	—	—	—	—	—	—	—	—	—	—	—	—	—	—	—	—	—
Nitrate reduction	—	—	—	—	—	—	—	—	—	—	—	—	—	—	—	—	—	—

Note: "+" is a positive reaction; "-" is a negative reaction.

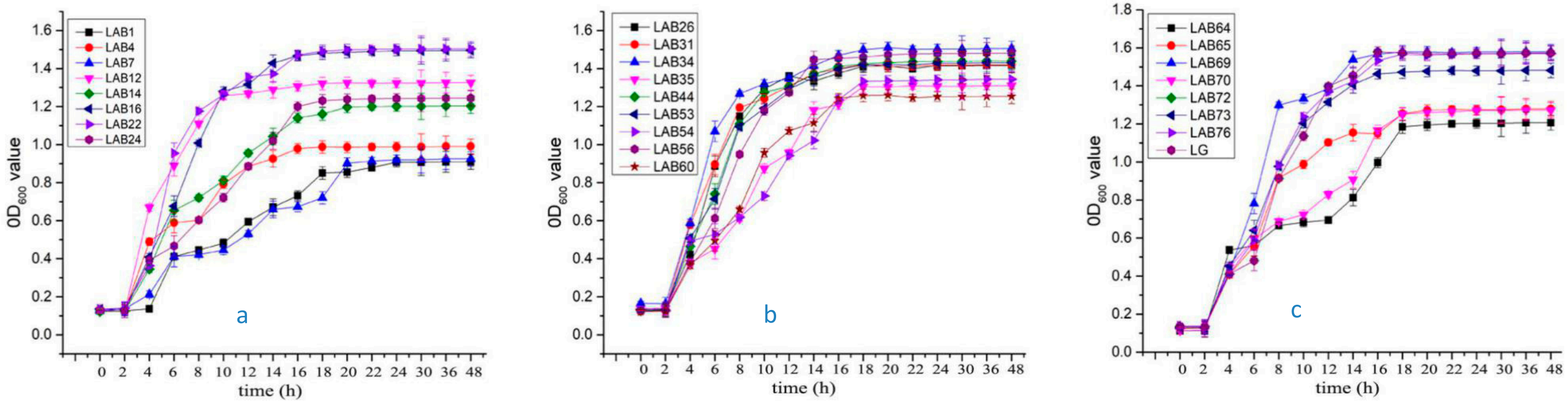

Figure S2. The growth curve of 24 selected strains of *Lactobacillus* within 48hs.

Note: a- The growth curve of strains LAB1, LAB4, LAB7, LAB12, LAB14, LAB16, LAB22, LAB24; b-The growth curve of strains LAB26, LAB31, LAB34, LAB35, LAB44, LAB53, LAB54, LAB56, LAB60; c- The growth curve of strains LAB64, LAB65, LAB69, LAB70, LAB72, LAB73, LAB76, LG.

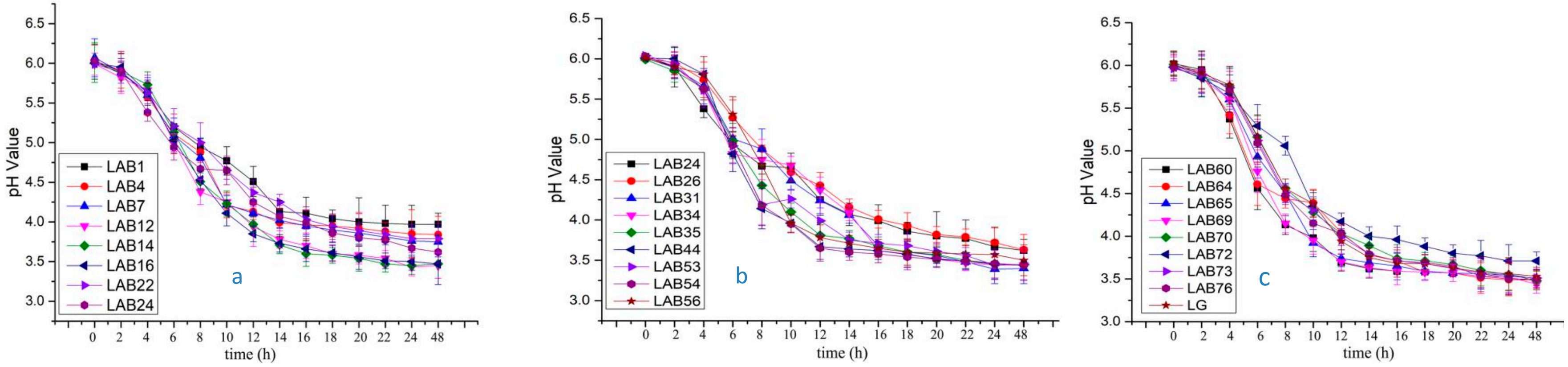

Figure S3. (a), (b) and (c) show the pH change of *Lactobacillus* during 48 h.

Note: a- The the pH change of strains LAB1, LAB4, LAB7, LAB12, LAB14, LAB16, LAB22, LAB24; b-The the pH change of strains LAB26, LAB31, LAB34, LAB35, LAB44, LAB53, LAB54, LAB56; c- The the pH change of strains LAB60, LAB64, LAB65, LAB69, LAB70, LAB72, LAB73, LAB76, LG.

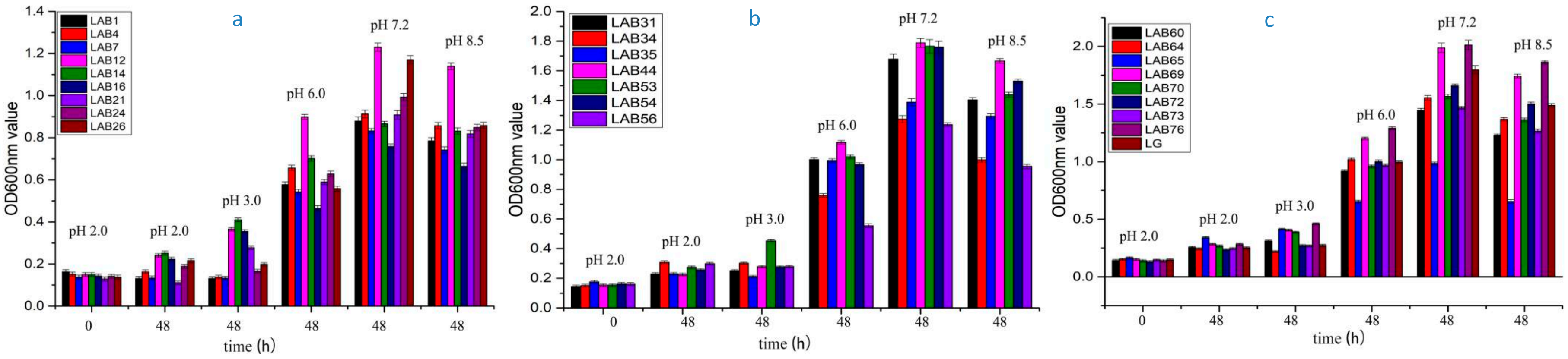

Figure S4. (a), (b) and (c) show the growth of 24 strains of *Lactobacillus* under five pH conditions.

Note: a- The growth of strains LAB1, LAB4, LAB7, LAB12, LAB14, LAB16, LAB22, LAB24 under five pH conditions.; b-The growth of strains LAB26, LAB31, LAB34, LAB35, LAB44, LAB53, LAB54, LAB56 under five pH conditions; c- The growth of strains LAB60, LAB64, LAB65, LAB69, LAB70, LAB72, LAB73, LAB76, LG under five pH conditions.

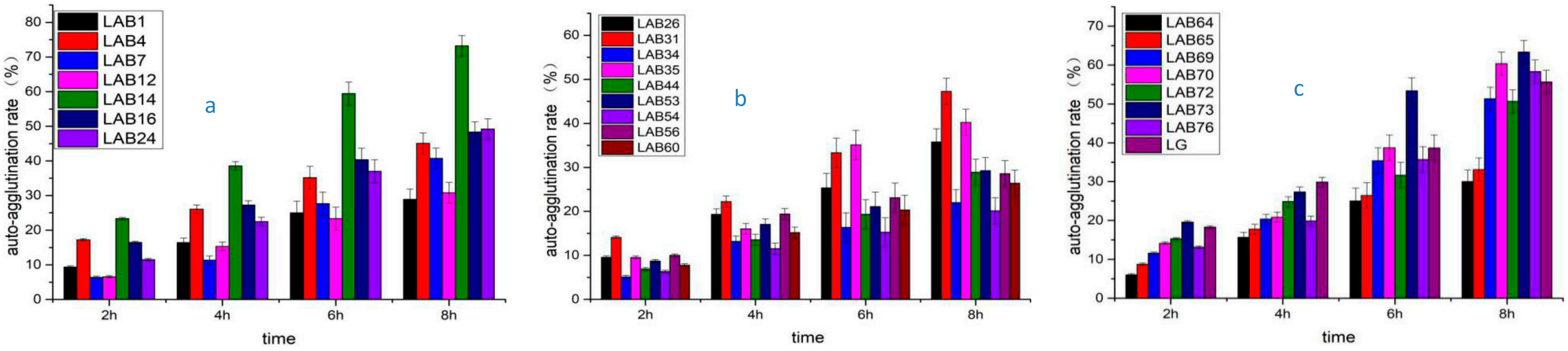

Figure S5. The self aggregation of 24 strains of *Lactobacillus*.

Note: a- The self aggregation of strains of LAB1, LAB4, LAB7, LAB12, LAB14, LAB16, LAB22, LAB24; b-The self aggregation of strains of LAB26, LAB31, LAB34, LAB35, LAB44, LAB53, LAB54, LAB56,LAB60; c: The self aggregation of strains of LAB64, LAB65, LAB69, LAB70, LAB72, LAB73, LAB76, LG.

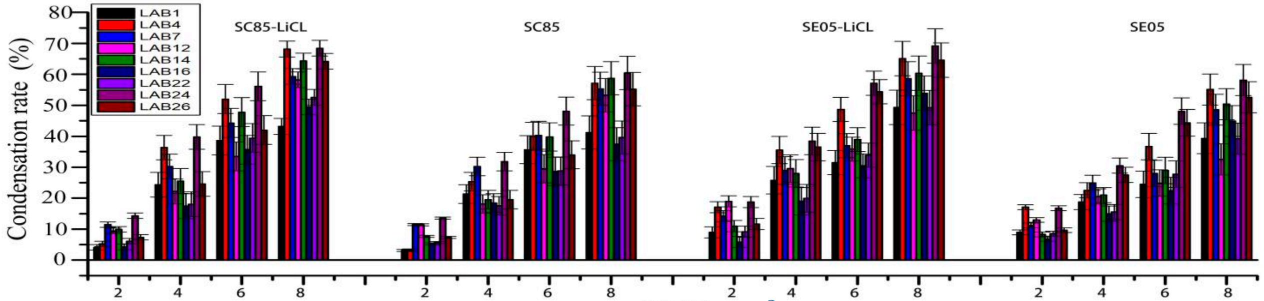

a

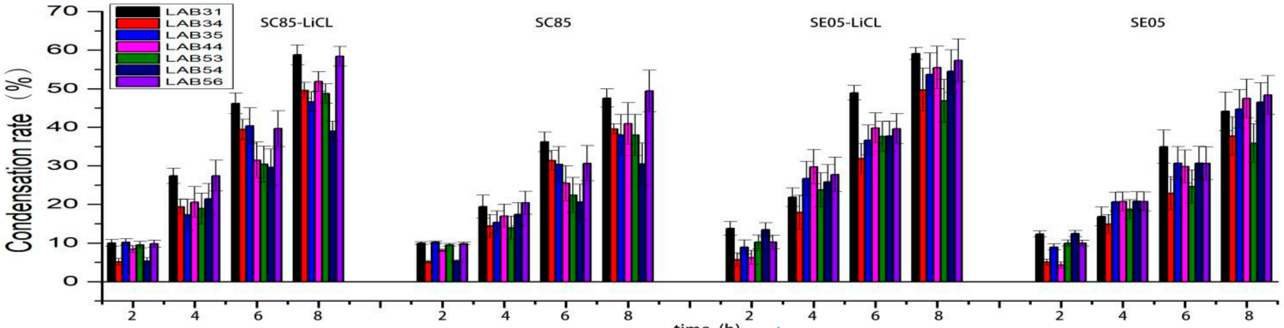

b

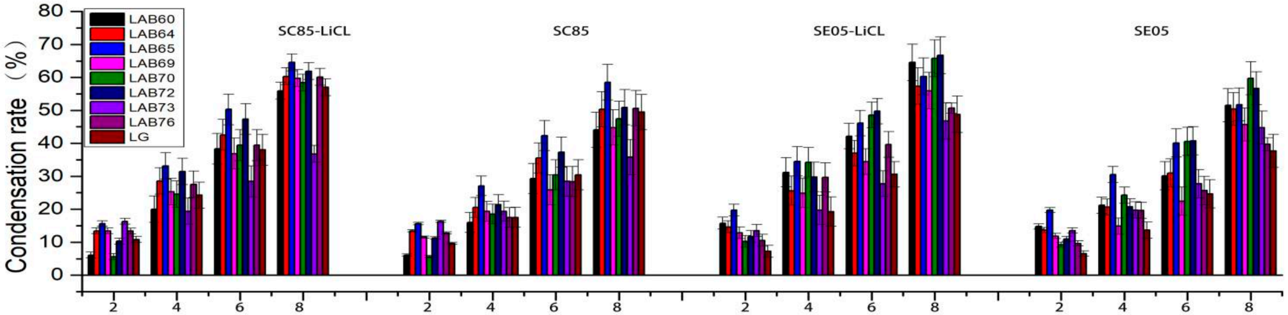

c

Figure S6. The agglutination effect of *Lactobacillus* treated with LiCL on two *Salmonella* strains.

Note: a, (b) and (c) indicated the agglutination effects of 24 selected *Lactobacillus* strains on two *Salmonella* strains after LiCL treatment.

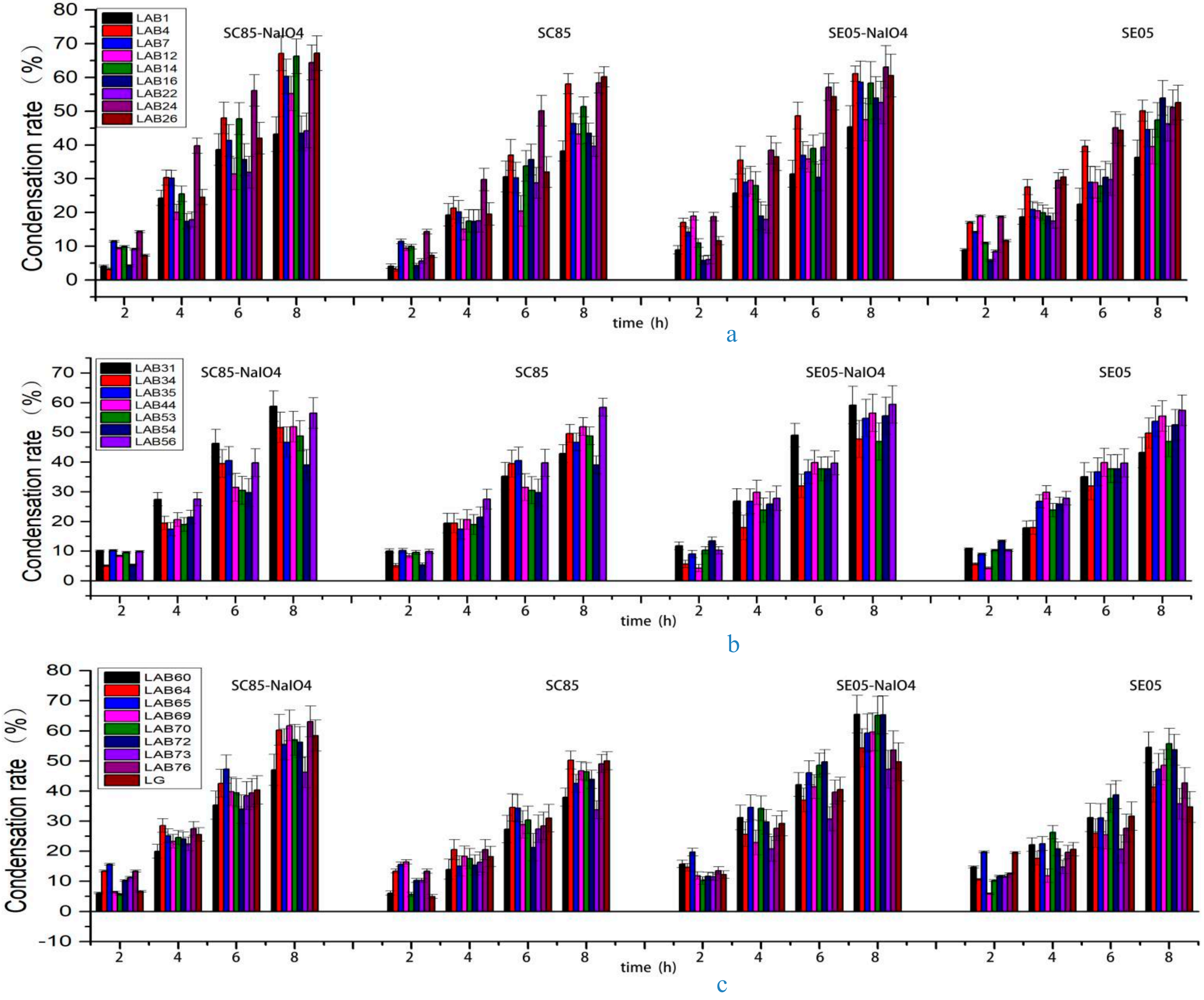

Figure S7. The agglutination effect of *Lactobacillus* treated with NaIO4 on two *Salmonella* strains.

Note: a, (b) and (c) indicated the agglutination effects of 24 selected *Lactobacillus* strains on two *Salmonella* strains after NaIO4 treatment.

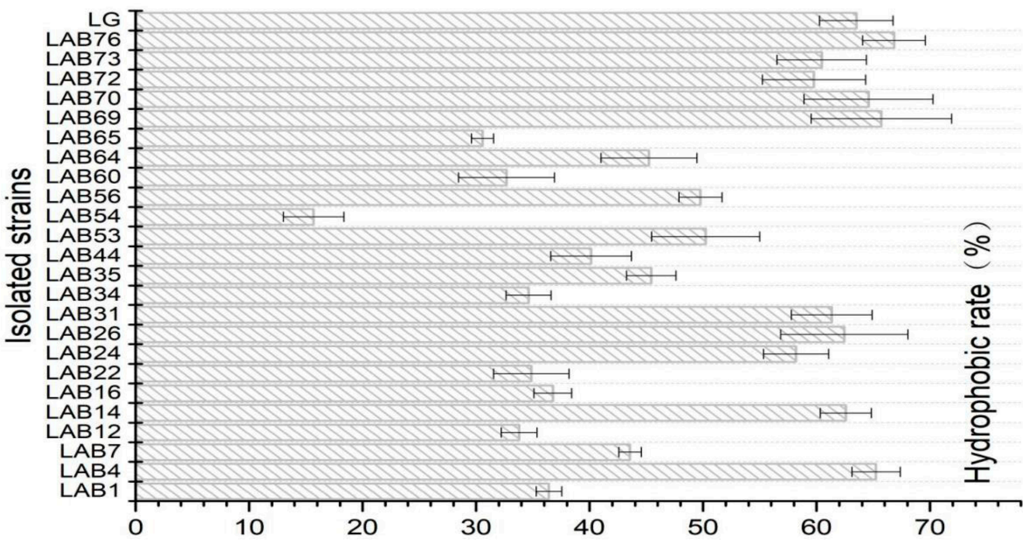

Figure S8. Hydrophobicity test results of 24 lactic acid bacteria strains.

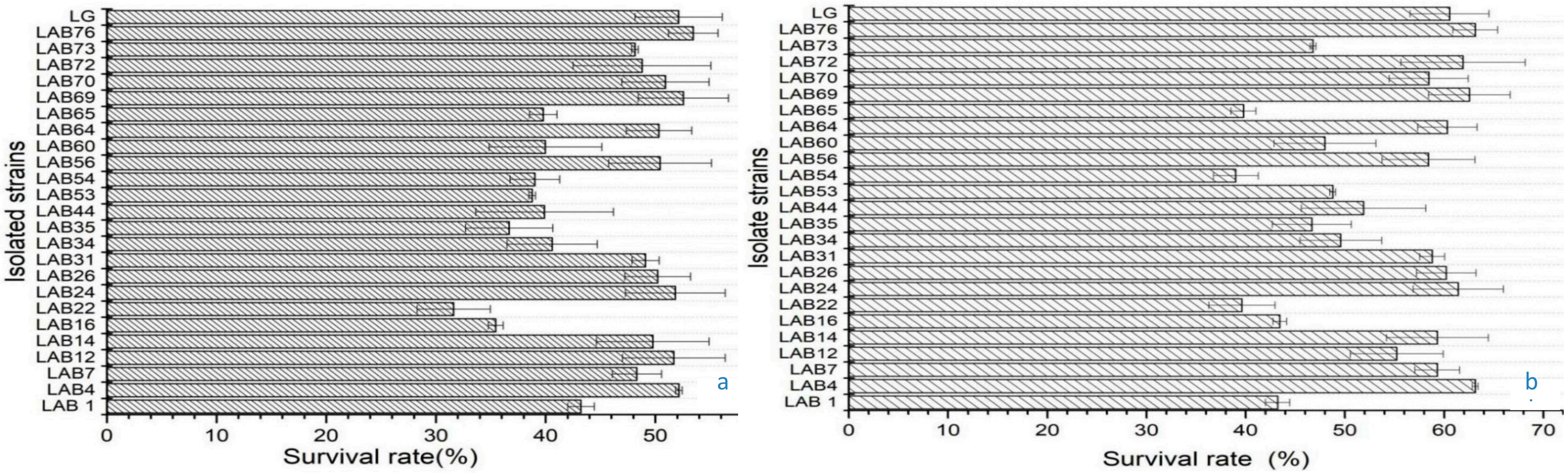

Figure S9. Tolerance test results of 24 strains of lactic acid bacteria in artificial simulated gastric fluid.

Note: a is the pepsin resistance test of 24 strains of *Lactobacillus* in the artificial simulated gastric juice; and b is the trypsin resistance test of 24 strains of *Lactobacillus* in the artificial simulated gastric juice.

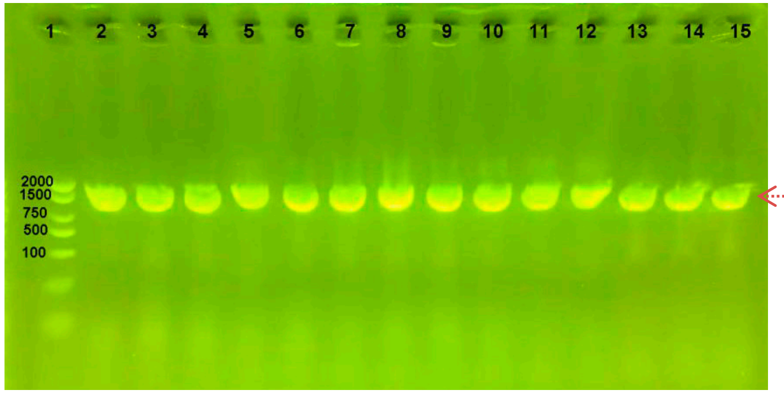

Figure S10-1. Electrophoresis results of *Lactobacillus* amplification products.

Note: Lane 1: Mark; Lane 2, 3: LAB4; Lane 4, 5:LAB31; Lane 6, 7:LAB35; Lane 8, 9: LAB44; Lane 10, 11: LAB53; Lane 12, 13: LAB54; Lane 14, 15: LAB60. The band fragment shown in the figure is 1500bp, which should appear [illegible]

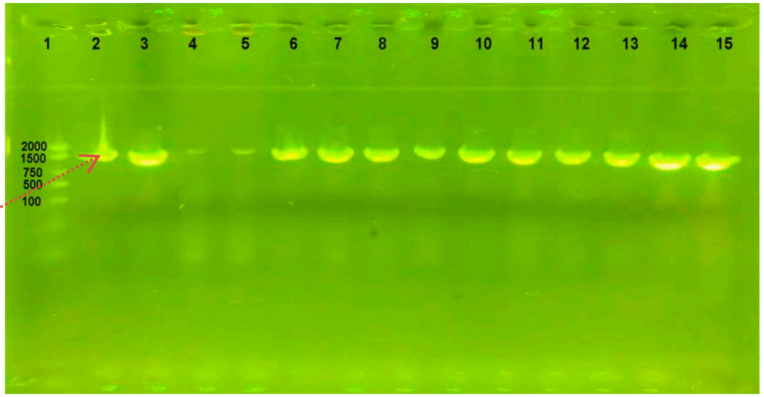

Figure S10-2. Electrophoresis results of *Lactobacillus* amplification products.

Note: Lane 1: Mark; Lane 2, 3: LAB65; Lane 4, 5:LAB64; Lane 6, 7:LAB69; Lane 8, 9: LAB72; Lane 10, 11: LAB73; Lane 12, 13: LAB76; Lane 14, 15: LAB79. The band fragment shown in the figure is 1500bp, which should appear
